# Bacteria in stable fly (Diptera: Muscidae) feces inform foraging decisions of conspecific flies

**DOI:** 10.1093/jme/tjag084

**Published:** 2026-06-13

**Authors:** Emmanuel Hung, Augustus Negraeff, Caelen Watson, Regine Gries, Pantea Zamani, Aryan Monfared, Cyan Luke, Gerhard Gries

**Affiliations:** Department of Biological Sciences, Simon Fraser University, Burnaby, BC, Canada; Department of Biological Sciences, Simon Fraser University, Burnaby, BC, Canada; Department of Biological Sciences, Simon Fraser University, Burnaby, BC, Canada; Department of Biological Sciences, Simon Fraser University, Burnaby, BC, Canada; Department of Biological Sciences, Simon Fraser University, Burnaby, BC, Canada; Department of Biological Sciences, Simon Fraser University, Burnaby, BC, Canada; Department of Biological Sciences, Simon Fraser University, Burnaby, BC, Canada; Department of Biological Sciences, Simon Fraser University, Burnaby, BC, Canada

**Keywords:** olfaction, filth flies, microbes, semiochemicals, attraction

## Abstract

Blood-feeding stable flies, *Stomoxys calcitrans* (L.), are reportedly attracted to conspecific feces. We investigated whether stable flies are attracted to fly feces-derived bacteria, and whether attraction varies with bacterial species and growth substrate, and the sex and reproductive status of foraging flies. We isolated seven bacterial species from fecal deposits of stable flies. In laboratory two-choice bioassays with paired adhesive-coated traps baited with sterile agar or bacteria-inoculated agar, the seven bacterial species in combination repelled flies. However, the bacterial congeners *Serratia marcescens* and *Serratia surfactantfaciens* attracted flies when tested singly. Analyses of the headspace volatiles of *S. marcescens* and *S. surfactantfaciens* by gas chromatography-mass spectrometry revealed near-identical odor profiles, which may explain their comparable attractiveness to flies. Flies were more attracted to *S*. *marcescens* grown on trypticase soy agar than to *S. marcescens* grown on bovine blood agar, likely due to substrate-dependent odor profiles. Feces-dwelling bacteria appear to function in the context of foraging rather than oviposition given that *S. marcescens* attracted both female and male stable flies, and both gravid and non-gravid females. That agar plates inoculated with all seven bacterial species failed to attract foraging flies could mean that the microbial community on agar did not sufficiently resemble that in fly feces, perhaps due to the limitations of culture-dependent isolation methods. Alternatively, additional feces-derived odors may also be necessary for fly attraction. Further investigation is warranted to identify the semiochemicals, and possibly other cues, produced by *S. marcescens* that make this bacterium distinctively attractive to stable flies.

## Graphical Abstract

**Graphical Abstract:**
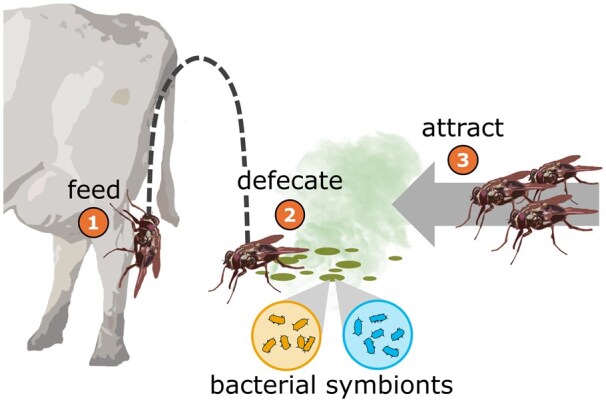

## Introduction

Foraging animals exploit informative, accurate, and easy-­to-assess cues to locate food (Dusenbery 1992). Foraging cues may originate from the resources being sought, or may be cues or signals associated with, or produced by, conspecifics (Leadbeater and Chittka 2007). Conspecific cues may help foraging individuals save time and energy locating resources (Wyatt 2003, Dall et al. 2005). The presence of conspecifics may signal the quality of a resource that was assessed, and deemed suitable, by these conspecifics (Prokopy and Roitberg 2001, Dall et al. 2005). In dipterans, conspecific cues may originate from the flies themselves (Schlein et al. 1984, Hung et al. 2026), from microbial symbionts, or from semiochemicals (message-bearing chemicals) the flies deposit on or near resources.

The long-known “fly factor” describes the phenomenon that food currently or previously fed on by flies attracts more foraging flies than the same food kept inaccessible to flies (Barnhart and Chadwick 1953). For example, nutrient resources visited by black blow flies, *Phormia regina*, house flies, *Musca domestica*, and *Drosophila* vinegar flies are more attractive to foraging conspecifics than similar resources that were not previously visited (Dethier 1955, Brodie et al. 2015, Keesey et al. 2016, Holl and Gries 2018). The cues which cause the fly factor phenomenon seem to be odors emanating from feces and regurgitate excreted by feeding flies (Keesey et al. 2016, Holl and Gries 2018). In blow flies, these fly-attracting odorants are thought to be metabolites produced by bacterial symbionts in the flies’ excreta (Uriel et al. 2020).

Stable flies, *Stomoxys calcitrans* (L.), are attracted to the odors emanating from fecal deposits of conspecifics (Carlson et al. 2000), reminiscent of the fly factor phenomenon observed in other fly taxa. Stable flies are obligate blood-feeding temporary ectoparasites of cattle and other livestock (Rochon et al. 2021). Haematophagous insects, including mosquitoes and tsetse flies, frequently defecate during or shortly after blood-feeding (Gee 1975, Stobbart 1977, Nijhout and Carrow 1978, Reisenman et al. 2011, Darrington 2015, Meraj et al. 2025). Immediate defecation after blood meals may mitigate thermal stress from ingested warm blood (Lahondère and Lazzari 2012), eliminate excess water and salts (Glasgow 1961, Gee 1975), and reduce the degree to which ingested blood meals impair flight (Glasgow 1961, Lehane 2005). Because stable flies likewise defecate immediately after a blood meal (Chen and Schleider 1966) and because they orient towards conspecific fecal deposits (Carlson et al. 2000), it is conceivable that these fecal deposits inform foraging conspecifics about the presence of a nearby host or on-host feeding site.

Like house flies and blow flies (Lam et al. 2007, Uriel et al. 2020), stable flies may exploit bacteria-derived semiochemicals as foraging and oviposition cues. Various bacteria affect stable fly behavior. For example, the microbial community composition in horse feces affects their attractiveness to ovipositing stable flies (Albuquerque and Zurek 2014). Bacterial isolates from stable fly larval habitats, stable fly eggs, and blow fly feces produce volatiles which inform oviposition decisions by gravid females (Romero et al. 2006, Hennig et al. 2024, Hung et al. unpubl. data). Similarly, *Staphylococcus* spp. in the cattle skin microbiome attract host-foraging stable flies (Nayani et al. 2023). Attraction of stable flies to fecal deposits of conspecifics, and stronger attraction to aged than to fresh feces (Carlson et al. 2000), potentially imply microbe-mediated stable fly attraction and a progressive shift in the microbial community and/or its metabolites.

If stable flies were to respond to feces-derived bacteria in a host-foraging rather than an oviposition context, both female and male flies would be expected to respond to these bacteria because both must blood-feed to reproduce (Anderson 1978, Chia et al. 1982). Because bacteria-emitted volatiles are metabolic by-products (Schmidt et al. 2015), foraging flies would be expected to respond most strongly when fecal bacterial symbionts are grown on substrates containing blood-meal-related nutrients. Here we tested three hypotheses (H): (1) foraging stable flies are attracted to semiochemicals of fly feces-derived bacteria; (2) stable fly attraction to bacterial semiochemicals varies with bacterial species and growth substrate; and (3) stable fly attraction to bacterial semiochemicals is unrelated to the sex and reproductive status of foraging flies.

## Materials and Methods

### Experimental Insects

To establish a laboratory colony, we field-collected stable flies from Eagle Acres Dairy and Pumpkin Patch (Langley, BC, Canada). Gravid flies were allowed to oviposit on squares (10 cm^2^) of moist black cotton fabric (Fabricana, Coquitlam, BC, Canada) placed on the ceiling of rearing cages. Eggs were water-rinsed off the fabric into plastic bins (35 × 30 × 15 cm, Dollarama, Montreal, QC, Canada) containing larval rearing media composed of NutraFin^®^ Basix tropical fish food (130 g, Rolf C. Hagen Inc., Montreal, QC, Canada), Rogers Edible wheat bran (500 g, Snow Cap Enterprises Ltd., Burnaby, BC, Canada), and spruce and fir wood shavings (200 g, Hyon Bedding Ltd., Prince George, BC, Canada) wetted and mixed thoroughly with 2 L of water. Pupae were transferred from rearing bins into metal mesh cages (45 cm × 45 cm × 45 cm; BioQuip^®^, Compton, CA, USA) in an ER-75 walk-in growth chamber (Bio Chambers Inc., Winnipeg, MB, 541 Canada) kept at 25°C, 60% RH, and a 14:10 h (light: dark) photoperiod. Once daily, adult flies were provisioned with bovine blood which was sourced from a local abattoir. To prevent coagulation, 10-L aliquots of fresh blood were mixed with 90 g of sodium citrate (99.5%; Bioshop, Burlington, ON, Canada) dissolved in 250 mL of distilled water. The flies tested in behavioral bioassays were 4- to 9-days-old (post-eclosion) and had been food-deprived 20 to 24 h. For bioassays, cold-sedated male and female flies were separated based on the following sex-specific characteristics: males have a larger dark spot than females on the two posterior-most sternites of their abdomen, and females have an ovipositor which extends distally when gentle pressure is applied on their abdomen using forceps.

### Collection and Isolation of Bacteria

**Fig. 1.**
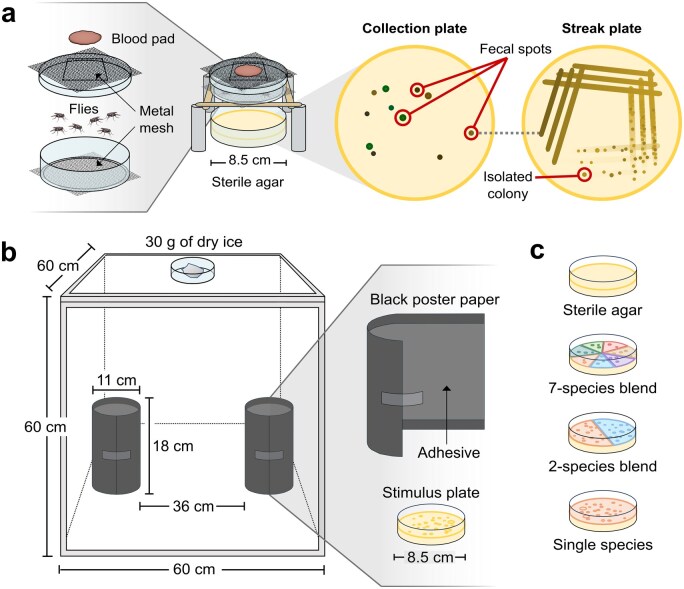
Graphical illustrations of experimental procedures and designs used to (a) collect and isolate bacteria in stable fly feces, (b) test behavioral responses of stable flies to bacteria in bioassays, and (c) present sterile agar plates and agar inoculated with single or multiple species of bacteria (7-species blend; 2-species blend). (a) Flies were confined in a petri dish modified to facilitate (i) feeding through metal mesh on a cotton pad soaked in warm citrated bovine blood, and (ii) defecation through metal mesh onto a sterile agar plate; three fecal spots on each agar plate were arbitrarily selected for streaking and 24-h incubation at 30°C. (b) A metal mesh cage was fitted with paired traps baited with a sterile agar plate (control) or an agar plate inoculated with a single bacterial species or multiple species grown on separate agar slices (see subpanel c). For each experimental replicate, 25 food-deprived flies were released into the cage, and fly captures on traps were recorded 30 min later.

Six groups of six female and six male flies were collected from a single cage cohort of 5-day-old flies. Each group was enclosed in a previously sterilized (70% ethanol) plastic petri dish (8.5 × 1.5 cm), with a mesh-covered square hole (4.5 × 4.5 cm) in the top through which flies could feed, and with the entire petri dish bottom replaced with metal mesh through which flies could defecate (Fig. 1a). The petri dish was placed on a sterilized stand (length: 11.5 cm, width: 13 cm, height: 5.5 cm) made from popsicle sticks (Rikkel Corp., Plano, TX, USA) and four inverted lidless vials (Fig. 1a). To initiate feces collection, the petri dish and stand were placed in a biological safety cabinet (NUAIRE Biological Safety Cabinets, Class II type A2), and a cotton round (Dollarama, Montreal, QC, Canada) soaked in warm (38°C) citrated bovine blood was placed on the metal mesh in the petri dish lid, allowing the flies to feed through the mesh (Fig. 1a). As soon as flies started blood-feeding, their fecal droplets were collected for 10 min on a sterile agar plate positioned beneath the petri dish. Expecting to collect feces-derived bacteria of diverse nutritional needs, feces were collected on Mueller-Hinton agar (MHA), nutrient agar (NA), and trypticase soy agar (TSA). To account for potential compositional changes of the flies’ fecal microbiota during blood digestion, fly feces were also collected during additional 10-min periods, starting at 60 and 240 min post-feeding. These time points were selected because digestion begins within 60 min of feeding, and flies begin excreting digested hemoglobin 240 min following ingestion (DeLoach and Spates 1979).

For each of the three time points (0, 60, and 240 min), feces were collected on 18 agar plates, two each of the three agar types. Three fecal spots from each plate were arbitrarily selected to be streak-plated and incubated 24 h at 30°C. Morphologically distinct colonies found on the resulting 54 streak plates were isolated by repeated re-streaking. All microbial work took place in a biological safety cabinet using aseptic techniques. Stock cultures of each isolate were preserved at −80°C in a 1:1:2 mixture of glycerol, distilled water, and broth culture. TSA or NA stock plates of bacteria (Supplementary Table 1) were kept at 4°C.

### Identification of Bacteria

Bacteria were identified by amplifying and sequencing the 16S rRNA gene (GENEWIZ, South Plainfield, NJ, USA) of isolated, morphologically distinct colonies and by comparing isolate sequences to sequences of identified microbes in the database of the National Center for Biotechnology Information GenBank (Bethesda, MD; http://www.ncbi.nlm.nih.gov/BLAST.cgi). A genus or species was considered a match if there was at least 99% coverage and 96% identity between a known sequence in the database and a sequenced isolate (Supplementary Table 1).

### General Experimental Design

In two-choice experimental replicates (‘bioassays’), flies were presented with paired cylindrical black poster paper traps (18 × 10 cm; Fig. 1b) whose interior surface was coated with Tanglefoot^®^ adhesive (Arbico Organics, Oro Valley, AZ, USA). The traps were positioned 36 cm apart in the center of a metal mesh cage (60 × 60 × 60 cm; BioQuip^®^) whose floor was lined with matte brown Kraft paper (NCR Corp., Duluth, GA, USA) and whose ceiling was replaced with fine white mesh netting (La Vogue, Amazon, Seattle, WA, USA) to increase overhead light transmission. The bioassay set-up was illuminated by a metal halide lamp (MT400D/BUD/HTL-BLUE, EYE Lighting Int., Mentor, OH, 554 USA) positioned 26.5 cm above the cage.

Traps were baited with a sterile agar control plate (8.5 cm in diameter) or an agar treatment plate that had been inoculated (by spread plating) with bacteria from a storage culture and incubated 24 h at 30°C prior to bioassays. To minimize potential side bias, the position of the treatment trap and the control trap was alternated between replicates.

To initiate a bioassay replicate, 25 flies were released into the bioassay cage and allowed 30 min to forage. Then, fly captures on traps were counted, and any flies remaining in the cage were cold-euthanized. Prior to the next bioassay, the air in the bioassay room with potentially lingering odor was vented out using an electric fan.

### Specific Experiments

#### H1: Foraging Stable Flies Are Attracted to Semiochemicals of Fly Feces-Derived Bacteria (Exps. 1 to 10)

This hypothesis was tested in experiments 1 to 10 (Table 1). To test whether semiochemical metabolites of bacteria in the feces of blood-fed stable flies attract conspecifics, flies in experiment 1 were offered a choice between a trap bait of sterile agar (agar plate diameter: 8.5 cm; control) and a trap bait of seven uniform agar slices (‘7-species blend’; Fig. 1c), all together amounting to the same total size as the control agar plate. Each slice was taken from a separate spread plate inoculated with a different bacterial species which had been incubated 24 h at 30°C on the same agar type (TSA or NA) on which it was originally isolated. The control plate presented the equivalent number of sterile TSA or NA slices in the same configuration as in the treatment plate.

**Table 1.** List of laboratory behavioral experiments (Exp.), the number of replicates (n) run, and the stimuli tested; all experiments tested 3- to 6-day-old female stable flies unless otherwise noted

Exp. (n)	Stimulus 1	Stimulus 2
**H1: Foraging stable flies are attracted to semiochemicals of fly feces-derived bacteria (Exps. 1 to 10)**		
**1 (15)^1^**	Sterile nutrient agar (NA) and sterile trypticase soy agar (TSA)	7-species blend^2^ on NA and TSA
**2 (15)**	Sterile NA and TSA	7-species blend^2^ on NA and TSA
**3 (15)**	Sterile bovine blood agar (BBA)	7-species blend^2^ on BBA
**4 (15)**	Sterile NA	*S. surfactantfaciens* on NA
**5 (15)**	Sterile TSA	*S. marcescens* on TSA
**6 (15)**	Sterile TSA	*N. circulans* on TSA
**7 (15)**	Sterile TSA	*P. huaxiensis* on TSA
**8 (15)**	Sterile TSA	*M. odoratus* on TSA
**9 (15)**	Sterile NA	*P. terrae* on NA
**10 (15)**	Sterile NA	*P. rettgeri* on NA
**H2: Stable fly attraction to bacterial semiochemicals varies with bacterial species and growth substrate (Exps. 11 to 16)**		
**11 (15)**	*S. marcescens* on TSA and *S. surfactantfaciens* on NA	*S. surfactantfaciens* on NA
**12 (15)**	*S. marcescens* on TSA and *S. surfactantfaciens* on NA	*S. marcescens* on TSA
**13 (15)**	*S. marcescens* on TSA	*S. surfactantfaciens* on NA
**14 (10)**	Sterile BBA	*S. marcescens* on BBA
**15 (10)**	*S. marcescens* on BBA	*S. marcescens* on TSA
**16 (7)**	*S. marcescens* on BBA	*S. surfactantfaciens* on BBA
**H3: Stable fly attraction to bacterial semiochemicals is unrelated to the sex and reproductive status of foraging flies (Exps. 17 to 19)**		
**17 (15)^3^**	Sterile TSA	*S. marcescens* on TSA
**18 (15)**	Sterile TSA	*S. marcescens* on TSA
**19 (15)^4^**	Sterile TSA	*S. marcescens* on TSA

1 Elevated levels of carbon dioxide served as an activation cue in all experiments except for experiment 1.

2 Seven uniform agar slices, each slice inoculated with a different bacterial species (Fig. 1c). The seven species, and the agar types on which they were cultured, are listed under ‘Stimulus 2’ in experiments 4 to 10.

3 Gravid >10-day-old female flies were tested in experiment 17.

4 Male flies were tested in experiment 19.

As CO_2_ activates stable fly foraging (Gatehouse and Lewis 1973, Warnes and Finlayson 1985), the 7-species blend and the sterile agar plate as trap baits were retested in experiment 2 but at elevated (rather than ambient) CO_2_ level produced by a 30-g piece of dry ice (liberating CO_2_) placed on the ceiling center of the bioassay cage (Table 1). As CO_2_ appeared to affect the responses of foraging bioassay flies (see Results), the protocol of all subsequent experiments included CO_2_. Considering that stable flies were sustained on bovine blood, which may contain essential nutrients for specific gut-dwelling bacteria, the 7-species blend was also grown on bovine blood agar (BBA) — which is 7% bovine blood — and tested as trap bait against sterile BBA (Exp. 3, Table 1).

To determine whether feces-derived bacterial species differed in their attractiveness to foraging stable flies, experiments 4 to 10 (Table 1) tested each bacterium on agar as a trap bait *versus* a sterile agar plate.

#### H2: Stable Fly Attraction to Bacterial Semiochemicals Varies With Bacterial Species and Growth Substrate (Exps. 11 to 16)

Given that *Serratia marcescens* (on TSA) and *Serratia surfactantfaciens* (on NA) attracted stable flies (see Results), we then tested for potential olfactory interaction between *S. marcescens* and *S. surfactantfaciens* on stable fly attraction. Multiple bacterial species may emit a semiochemical blend that is more attractive to flies than the emissions of a single species (Uriel et al. 2020). Thus, flies were offered a choice between an agar plate of two equal slices — one inoculated with *S. marcescens* on TSA and the other with *S. surfactantfaciens* on NA (‘2-species blend’; Fig. 1c) — and a plate of either *S. marcescens* on TSA (Exp. 11) or *S. surfactantfaciens* on NA (Exp. 12). To further determine the species most attractive to flies, we offered flies a choice between two plates — one with *S. marcescens* on TSA and the other with *S. surfactantfaciens* on NA (Exp. 13). To ascertain whether the attractiveness of *S. marcescens* was dependent upon the type of agar provided as bacterial growth medium, flies in experiments 14 to 16 (Table 1) were offered a choice between (i) BBA plates kept sterile or inoculated with *S. marcescens* (Exp. 14), (ii) TSA plates kept sterile or inoculated with *S. marcescens* (Exp. 15), and (iii) BBA plates inoculated with *S. marcescens* or *S. surfactantfaciens* (Exp. 16). Due to limited supply of sterile bovine blood, only 10 (instead of 15) replicates were run for experiments 14 to 15, and seven for experiment 16.

#### H3: Stable Fly Attraction to Bacterial Semiochemicals Is Unrelated to the Sex and Reproductive Status of Foraging Flies (Exps. 17 to 19)

To determine whether stable fly attraction to bacterial semiochemicals varies with fly sex and reproductive status, three groups of flies were offered a choice between a sterile TSA plate and a TSA plate inoculated with *S. marcescens:* gravid >10-day-old females (Exp. 17), non-gravid 3- to 6-day-old females (Exp. 18), and 3- to 6-day-old males (Exp. 19) (Table 1).

### Collection of *Serratia* Headspace Volatiles and Gases

As *S*. *marcescens* (on TSA) and *S*. *surfactantfaciens* (on NA) attracted foraging stable flies (see Results), we proceeded to collect their headspace volatiles for comparative analysis. To this end, 12 TSA plates inoculated with *S*. *marcescens*, and 12 NA plates inoculated with *S*. *surfactantfaciens*, were incubated separately 24 h at 30°C. The TSA plates, or NA plates, were then placed into a glass chamber (19 × 29.5 cm) connected to a vacuum pump (Neptune Dyna-pump, Neptune Products Inc., Toronto, ON, Canada). The pump drew charcoal-filtered air (1 L min^−1^ for 24 h) through the chamber and a glass column (6 × 150 mm) containing 200 mg of manufacturer-preconditioned Porapak-Q™ (50–80 mesh; Waters Associates, Milford, MA, USA), 200 mg of Tenax TA (35–60 mesh; Chromatographic Specialties Inc., Brockville, ON, Canada), and 500 mg of Carbosieve™ (60–80 mesh; Supelco^®^, Bellefonte, PA, USA) adsorbents. Headspace volatiles captured on these adsorbents were desorbed with a pentane/ether rinse (1/1; 2 mL), concentrated to 0.2 mL, and stored at 4°C prior to analyses.

As an alternative method for collection of bacterial headspace volatiles, a spread plate inoculated with either *S*. *marcescens* (on TSA) or *S*. *surfactantfaciens* (on NA) was placed into a 1-L amber glass jar capped by an I-Chem™ closed-top lid and fitted with a white silicone septum (Fisher Scientific, Ottawa, ON, Canada). Then, a solid phase microextraction (SPME) fiber (75 μm CAR/PDMS, Fused Silica 23Ga; Supelco^®^), which served to adsorb airborne volatiles, was inserted through the septum such that its terminus end resided 12 cm above the agar surface. The SPME fiber was kept in the jar for 5 min before it was withdrawn.

We used a Q-Trak Indoor Air Quality Monitor (Model 7575; TSI Inc., Shoreview, MN, USA) and a MultiRAE Wireless Portable Six-Gas Monitor (Honeywell, Charlotte, NC, USA) to quantify carbon dioxide (CO_2_) and ammonia (NH_3_) gas emission, respectively, from spread plates of each of the feces-derived bacterial species.

### Chemical Analyses of *Serratia* Headspace Volatiles

Aliquots of headspace volatile extract, and volatiles thermally desorbed from SPME fibers, were analyzed by gas chromatography–mass spectrometry (GC–MS) using an Agilent 5977 A Series MDS coupled to an Agilent 7890B GC (Agilent Technologies Inc., Santa Clara, CA, USA). The instrument was operated in full-scan electron ionization mode and fitted with a DB-5 GC-MS column (30 m × 0.25 mm ID, film thickness 0.25 μm; Agilent Technologies). The injector port, MS source, and MS quadrupole were set to 250, 230, and 150°C, respectively. Helium was used as a carrier gas (39.7 cm s^-1^; 5:1 split ratio), with the following temperature program: 40°C (held 5 min), 10°C · min^−1^ to 280°C (held 10 min). Compounds were identified by comparing their mass spectra and retention indices with those of authentic standards that were either purchased or synthesized in the Gries-laboratory (Supplementary Table 2, Gries et al. 2002).

### Statistical Analysis

Data were analyzed with R statistical software (4.5.0, R Core Team 2025) using RStudio (2025.05.0 + 496, RStudio Team 2025) and the packages *tidyr* and *dplyr*. We used generalized linear models with a binomial distribution and a logit link function to compare an intercept-only model against a null model with a likelihood ratio test (LRT). In each experiment, this allowed us to determine significant differences (*P *< 0.05) in the proportion of flies captured on treatment and on control traps, with the total proportion of flies captured on both traps being 1.0. For each LRT, the degrees of freedom (df) were equal to 1 — the difference in the number of parameters between the two models (Wilks 1938).

## Results

### Identification of Bacteria

Seven bacterial species were isolated from stable fly feces and identified: *Serratia marcescens*, *Serratia surfactantfaciens*, *Niallia circulans*, *Providencia huaxiensis*, *Myroides odoratus*, *Proteus terrae*, and *Providencia rettgeri* (Supplementary Table 1).

Of these species, *S. marcescens*, *N. circulans*, *P. huaxiensis*, and *M. odoratus* were isolated and cultured on TSA, whereas *S. surfactantfaciens*, *P. terrae*, and *P. rettgeri* were isolated and cultured on NA. The bacteria *S. surfactantfaciens*, *P. huaxiensis*, and *P. rettgeri* were also isolated on MHA, but were more easily cultured on NA or TSA.

#### H1: Foraging Stable Flies Are Attracted to Semiochemicals of Fly Feces-Derived Bacteria (Exps. 1 to 10)

**Fig. 2.**
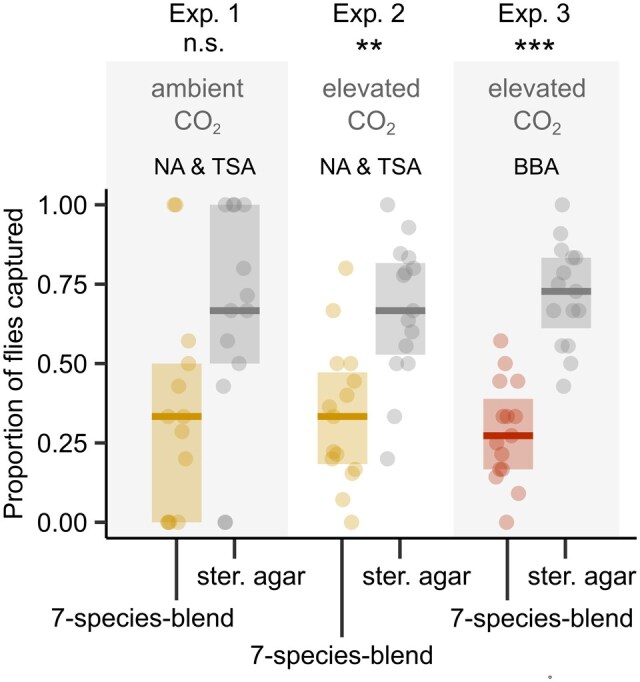
Effects of bacteria isolated from stable fly feces and grown on agar as a trap bait on attraction of stable flies in experiments 1 to 3 (*N *= 15 each). Paired adhesive traps (Fig. 1b) were baited with either a control plate of sterile (ster.) agar [nutrient agar (NA), trypticase soy agar (TSA), bovine blood agar (BBA)] or a treatment plate of seven uniform agar slices, each slice inoculated with a different bacterial strain isolated from stable fly feces [*Myroides odoratus* (TSA), *Niallia circulans* (TSA), *Proteus terrae* (NA), *Providencia huaxiensis* (TSA), *Providencia rettgeri* (NA), *Serratia marcescens* (TSA), and *Serratia surfactantfaciens* (NA)] (‘7-species blend’; Fig. 1c). The control plate presented the equivalent number of sterile TSA or NA slices in the same configuration as in the treatment plate. In each bioassay replicate, 25 food-deprived female flies were released into the bioassay cage and fly captures on traps were recorded 30 min later. To stimulate host-foraging behaviors of flies, a 30-g piece of dry ice (liberating CO_2_) was placed on the bioassay cage ceiling (Exps. 2 to 3). Dots represent the data of individual replicates, whereas the thick horizontal line within boxplots indicates the mean proportional response of flies. Asterisks (*) denote a significant effect of a stimulus on fly captures (** *P *< 0.01, *** *P *< 0.001; ‘n. s.’ = not significant (*P *> 0.05); likelihood ratio tests).

At ambient CO_2_ level (dry ice absent), flies were equally attracted to traps baited with seven slices of NA/TSA — each inoculated with a different bacterial species (‘7-species blend’) — and to traps baited with an equivalent combination of sterile NA and TSA (Exp. 1: *F *= 4.009, df = 1, *P *= 0.0684; Fig. 2). At elevated CO_2_ level (dry ice present), fewer flies were captured on traps baited with the 7-species blend on NA/TSA than on traps baited with sterile NA/TSA (Exp. 2: *F *= 13.546, df = 1, *P *= 0.0025; Fig. 2). Furthermore, at elevated CO_2_ level (dry ice present), fewer flies were captured on traps baited with the 7-species blend on bovine blood agar (BBA) than on sterile BBA-baited traps (Exp. 3: *F *= 24.777, df = 1, *P *= 0.0002; Fig. 2). All data combined indicate that CO_2_ levels affect the flies’ foraging responses, and that the presence of one or more bacteria (or their metabolites) in the 7-species blend is repellent to flies.

**Fig. 3.**
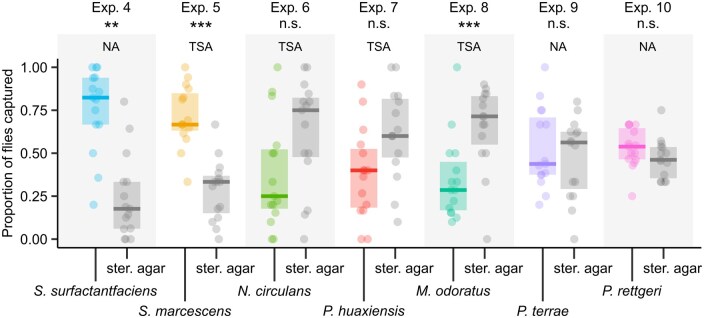
Effects of bacteria isolated from stable fly feces and grown on agar as a trap bait on attraction of stable flies in experiments 4 to 10 (*N *= 15 each). Paired adhesive traps (Fig. 1b) were baited with either a sterile (ster.) agar control plate or an agar treatment plate inoculated with one of seven bacterial isolates. In each bioassay replicate, 25 food-deprived and CO_2_-activated female flies were introduced into the bioassay cage and fly captures on traps were recorded 30 min later. Dots represent the data of individual replicates, whereas the thick horizontal line within boxplots indicates the mean proportional response of flies. Asterisks (*) denote a significant effect of a stimulus on fly captures (** *P *< 0.01, *** *P *< 0.001; ‘n. s.’ = not significant (*P *> 0.05); likelihood ratio tests).

When paired traps were baited with sterile agar or agar inoculated with a single bacterial species (Exps. 4 to 10), traps baited with *S. surfactantfaciens* on NA (Exp. 4), or *S. marcescens* on TSA (Exp. 5), captured more flies than sterile agar-baited traps (Exp. 4: *F *= 12.756, df = 1, *P *= 0.0031; Exp. 5: *F *= 23.942, df = 1, *P *= 0.0002; Fig. 3). In contrast, traps baited with *M. odoratus* on TSA captured fewer flies than sterile TSA-baited traps (Exp. 8: *F *= 12.674, df = 1, *P *= 0.0031; Fig. 3). Captures were unaffected by the absence or presence of bacteria when the bacterium tested was *N. circulans* on TSA (Exp. 6: *F *= 2.636, df = 1, *P *= 0.1268), *P. huaxiensis* on TSA (Exp. 7: *F *= 1.803, df = 1, *P *= 0.2007), *P. terrae* on NA (Exp. 9: *F *= 0.0188, df = 1, *P *= 0.8929), or *P. rettgeri* on NA (Exp. 10: *F *= 2.515, df = 1, *P *= 0.1351; Fig. 3).

#### H2: Stable Fly Attraction to Bacterial Semiochemicals Varies With Bacterial Species and Growth Substrate (Exps. 11 to 16)

**Fig. 4.**
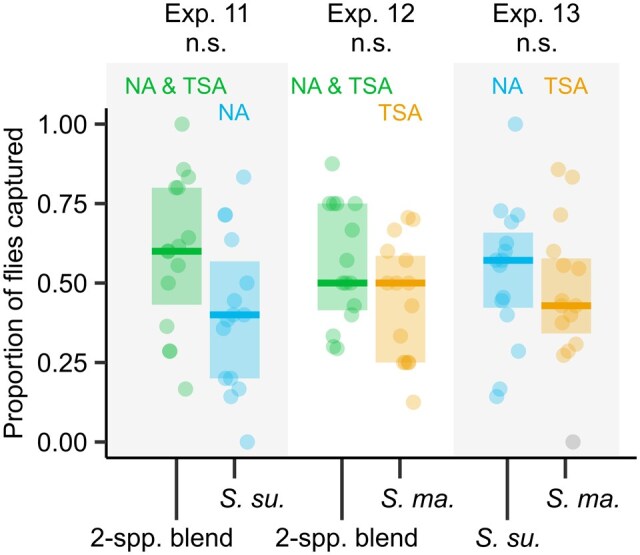
Effects of bacteria isolated from stable fly feces and grown on agar as a trap bait on attraction of stable flies in experiments 11 to 13 (*N *= 15 each). Paired adhesive traps (Fig. 1b) were baited either with two uniform agar slices, one inoculated with *Serratia marcescens* and the other with *Serratia surfactantfaciens* (‘2-spp. blend’; Fig. 1c), or with an agar plate inoculated with *S. surfactantfaciens* (*S. su*.) (Exp. 11) or *S. marcescens* (*S. ma*.) (Exp. 12). In experiment 13, paired traps were baited with *S. surfactantfaciens* or *S. marcescens*. In each bioassay replicate, 25 food-deprived and CO_2_-activated female flies were introduced into the bioassay cage and fly captures were recorded 30 min later. Dots represent the data of individual replicates, whereas the thick horizontal line within boxplots indicates the mean proportional response of flies; ‘n. s.’ = not significant (*P* > 0.05) denotes that there was no significant effect of a stimulus on fly captures (likelihood ratio tests).

Traps baited with both *S. surfactantfaciens* and *S. marcescens* grown on separate slices of NA and TSA (‘2-species blend’; Fig. 1c) captured as many flies as paired traps baited with either *S. surfactantfaciens* on NA (Exp. 11: *F *= 1.802, df = 1, *P *= 0.2008; Fig. 4) or *S. marcescens* on TSA (Exp. 12: *F *= 0.446, df = 1, *P *= 0.5151; Fig. 4). Similarly, fly captures on paired traps did not differ when traps were baited with either *S. surfactantfaciens* on NA or *S. marcescens* on TSA (Exp. 13: *F *= 0.060, df = 1, *P *= 0.8095; Fig. 4).

**Fig. 5.**
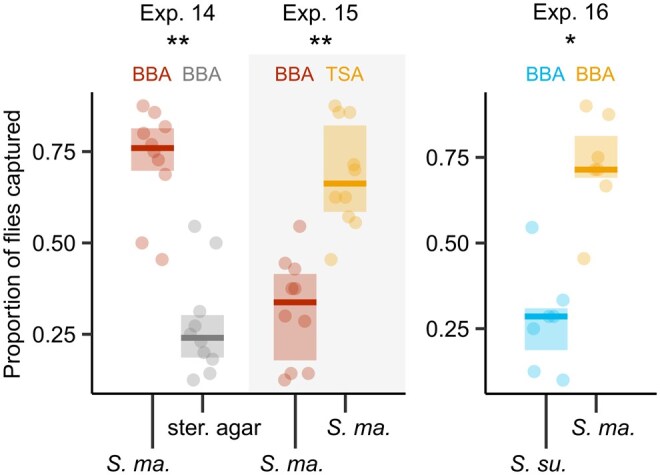
Effects of agar type on growth of bacteria isolated from stable fly feces and their attractiveness to stable flies in experiments 14 to 15 (*N *= 10 each) and 16 (*N *= 7). Paired adhesive traps were baited with (i) sterile (ster.) bovine blood agar (BBA) or BBA inoculated with *Serratia marcescens* (*S. ma*) (Exp. 14), (ii) BBA or trypticase soy agar (TSA) each inoculated with *S. marcescens* (Exp. 15), and (iii) BBA inoculated with either *S. marcescens* or *S. surfactantfaciens* (*S. su*.) (Exp. 16). In each bioassay replicate, 25 food-deprived and CO_2_-activated female stable flies were introduced into the bioassay cage and fly captures on adhesive traps were recorded 30 min later. Dots represent the data of individual replicates, whereas the thick horizontal line within boxplots indicates the mean proportional response of flies. Asterisks (*) denote a significant effect of a stimulus on fly captures (* *P* < 0.05, ** *P* < 0.01; likelihood ratio tests).

The relative attractiveness of *S. surfactantfaciens* and *S. marcescens* to flies was affected by the agar provisioned for bacterial growth. Whereas *S. marcescens* grown on BBA as a trap bait afforded more fly captures than sterile BBA as a trap bait (Exp. 14: *F *= 21.964, df = 1, *P *= 0.0011; Fig. 5), *S. marcescens* grown on TSA afforded more fly captures than *S. marcescens* grown on BBA (Exp. 15: *F *= 14.053, df = 1, *P *= 0.0046; Fig. 5). When *S. surfactantfaciens* and *S. marcescens* were grown separately on BBA as trap baits*, S. marcescens* afforded more fly captures than *S. surfactantfaciens* (Exp. 16: F = 10.445, df = 1, *P *= 0.0179; Fig. 5).

#### H3: Stable Fly Attraction to Bacterial Semiochemicals Is Unrelated to the Sex and Reproductive Status of Foraging Flies (Exps. 17 to 19)

**Fig. 6.**
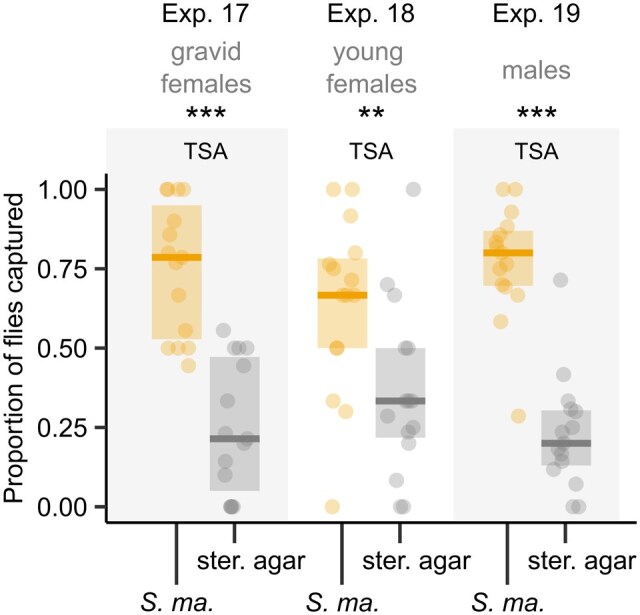
Effects of sex and physiological state (gravid or not) of stable flies on fly captures in experiments 17 to 19 (*N *= 15 each). Paired adhesive traps were baited with sterile (ster.) trypticase soy agar (TSA) or TSA inoculated with the bacterium *Serratia marcescens* (*S. ma*.) isolated from stable fly feces. In each bioassay replicate, 25 food-deprived and CO_2_-activated flies were introduced into the bioassay cage and captures of gravid females (Exp. 17), young females (Exp. 18), and males (Exp. 19) on paired traps (Fig. 1b) were recorded 30 min later. Dots represent the data of individual replicates, whereas the thick horizontal line within boxplots indicates the mean proportional response of flies. Asterisks (*) denote a significant effect of a stimulus on fly captures (** *P* < 0.01, *** *P* < 0.001; likelihood ratio tests).

All data below indicate that *S. marcescens* on TSA is attractive to all flies irrespective of their sex and reproductive state. Compared to traps baited with sterile TSA, paired traps baited with *S. marcescens* on TSA captured more gravid female flies (Exp. 17: *F *= 19.681, df = 1, *P *= 0.0006), non-gravid female flies (Exp. 18: *F *= 9.703, df = 1, *P *= 0.0076), and male flies (Exp. 19: *F *= 22.003, df = 1, *P *= 0.0003; Fig. 6).

### Headspace Volatiles and Gases Emitted by *S. surfactantfaciens* and *S. marcescens*

**Fig. 7.**
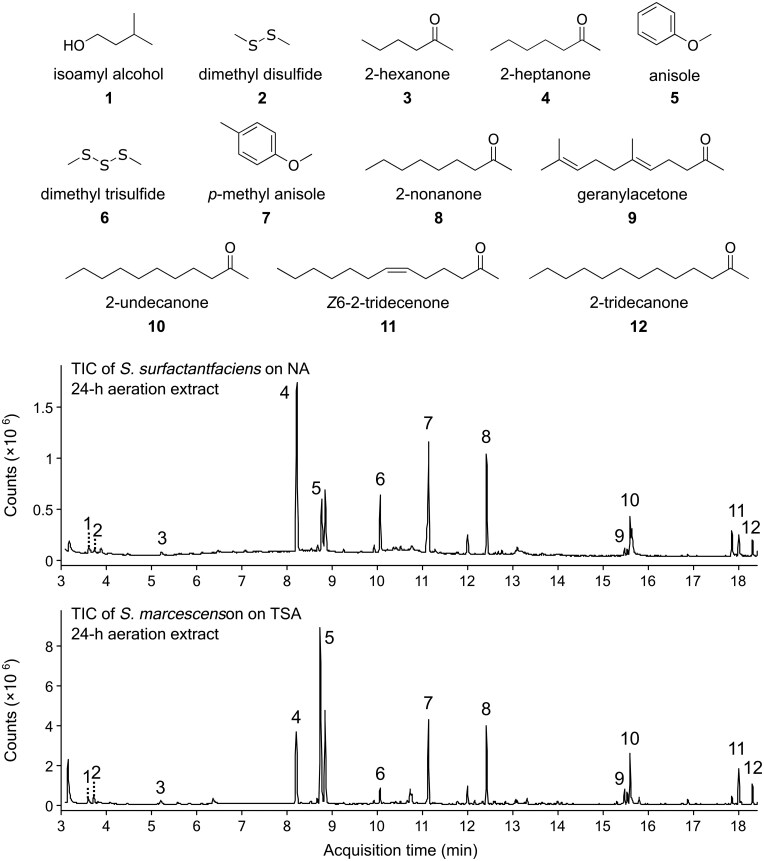
Total ion chromatograms (TICs) of volatiles in the headspace of *Serratia marcescens* (*S. mar*.) and *S. surfactantfaciens* (*S. sur*.) isolated from stable fly feces and grown on trypticase soy agar (TSA) and nutrient agar (NA). Headspace volatile extracts were analyzed by gas chromatography–mass spectrometry (GC–MS; Agilent 7890B GC coupled to a 5977 A Series MSD, DB-5 MS column). Numbers on TIC peaks correspond to numbers below chemical structures. Note the similarity in the volatile profiles.

The headspace volatile blend of *S. surfactantfaciens* and *S. marcescens* was nearly identical, comprising more than 12 volatiles: isoamyl alcohol, dimethyl disulfide, 2-hexanone, 2-heptanone, anisole, dimethyl trisulfide, *p*-methyl anisole, 2-nonanone, geranylacetone, 2-undecanone, *Z*6-2-tridecenone, 2-tridecanone (Fig. 7).

When grown on agar, all seven feces-derived bacteria, including *S. surfactantfaciens* and *S. marcescens*, emitted CO_2_ and NH_3_ gases (Supplementary Table 3).

## Discussion

Our data support the hypothesis that stable flies are attracted to certain bacteria in the feces of conspecific flies. The seven bacterial species in combination, each isolated from stable fly feces and grown on the agar type on which it was collected, failed to attract and even repelled flies (Fig. 2). However, two bacteria — *S. marcescens* and *S. surfactantfaciens —* attracted flies when tested singly (Fig. 3). When both species were grown on BBA, *S. marcescens* was more attractive to flies than *S. surfactantfaciens* (Fig. 5). Counter to our prediction, *S. marcescens* grown on BBA was less attractive to flies than *S. marcescens* grown on TSA (Fig. 5). Together, our data suggest that bacteria produce species-specific semiochemical blends, with blend compositions modulated by the growth substrate of the bacteria.

Our interpretation that semiochemicals of feces-dwelling bacteria function in the context of foraging, rather than oviposition, was supported by bioassay data showing that *S. marcescens* attracted stable flies regardless of their sex and reproductive status (Fig. 6). The effect of this bacterial foraging cue distinctively differs from that of the aggregation pheromone emitted by sand flies (Schlein et al. 1984) and from that of the chemically mediated ‘invitation effect’ in mosquitoes and black flies (Alekseev et al. 1977, Cavanagh and Townson 1986, McCall and Lemoh 1997, Charlwood et al. 2014). Sandflies, mosquitoes, and black flies must be physically present and actively feeding to attract conspecifics. In contrast, the bacterial foraging cue of stable flies remains attractive to stable flies even long after conspecifics have left. As such, this cue is reminiscent of the fly factor phenomenon reported in other fly taxa (see above; Dethier 1955, Carlson et al. 2000, Holl and Gries 2018). As stable flies typically blood-feed 1 to 2 times per day (Harris et al. 1974, Berry and Campbell 1985), and defecate immediately after feeding (Chen and Schleider 1966), fecal deposits and their odors may accumulate over time in close vicinity to suitable hosts. That stable fly feces attract stable flies for up to 30 days (Carlson et al. 2000) implies that signaling microbes remain viable in fecal deposits for a long time or that amassed (microbial) metabolites continue to emanate from fecal deposits even after microbial activity has ceased.

There are multiple possible explanations for the unexpected repellency of the 7-species blend (Fig. 2). Firstly, the repellency may have been caused, in part, by *M. odoratus* emitting off-putting odors that masked the attractive odors emitted by *S. marcescens* and *S. surfactantfaciens* (Fig. 3). Secondly, the 7-species blend may have been missing key bacteria essential for stable fly attraction. Standard culture techniques often fail to capture complete microbial communities because culture-dependent isolation methods are biased due to difficulties in replicating metabolic and physiological requirements of microbes in vitro (Amann et al. 1995, Hugenholtz et al. 1998, Martiny 2019). If only a fraction of the microbes in stable fly feces were represented by the 7-species blend, inadvertent omission of microbial species could have altered the attractiveness of this blend. The effect of this potential alteration could have been further compounded if stable fly attraction were to rely on odors from multiple key bacteria interacting synergistically, as shown in blow flies (Uriel et al. 2020). Thirdly, because we used equal slices of each bacterial species to present our 7-species blend, the proportional composition of the bacterial community and their associated metabolites were likely distorted, possibly affecting fly attraction. Lastly, growing bacteria on agar, rather than on fly feces, may have caused substrate-dependent qualitative and quantitative shifts in the metabolites produced (Lysyk et al. 2002, Basso et al. 2022, Rajendran et al. 2024).

The attractiveness of *S. marcescens* to stable flies is perplexing because *S. marcescens* has documented adverse effects on insect hosts, including stable flies (Watson and Petersen 1991, Lysyk et al. 2002), green bottle flies, *Lucilia sericata* (O’Callaghan et al. 1996), house flies (Benoit et al. 1990, Li et al. 2023), and various tsetse flies (Poinar et al. 1979, Kaaya and Darji 1989). Certain strains of *S. marcescens* produce toxins that cause cell lysis (Kurz et al. 2003, Trejo-López et al. 2022) and chitinase enzymes that break down chitin in the insects’ cuticle (Tao et al. 2022). However, the pathogenicity of *S. marcescens* varies with growth medium (Lysyk et al. 2002) and strain (O’Callaghan et al. 1996). Symbiotic *S. marcescens* (and *S. surfactantfaciens*) strains may potentially even aid stable fly survival by expressing antimicrobial compounds that suppress fungal growth and prevent fungal infection (Su et al. 2016, Trejo-López et al. 2022, Xin et al. 2023).

*Serratia marcescens* was isolated here from stable fly feces but it is widespread and modifies fly behavior in multiple ­contexts. It was previously isolated from house fly larvae (Li et al. 2023) and adult stable flies (Lysyk et al. 1999, Castro et al. 2007), apple maggot flies, *Rhagoletis pomonella* (Lauzon et al. 2003), and tsetse flies (Ibrahim et al. 2022). *Serratia marcescens* isolated from natural substrate infested with 3rd instar stable fly larvae deterred oviposition by gravid female flies (Romero et al. 2006). In contrast, *S. marcescens* isolated from stable fly eggs induced oviposition by gravid female stable flies (Hung et al. unpubl. data), as did a strain isolated from the excreta of black blow flies, *Phormia regina* (Hennig et al. 2024). In the current study, *S. marcescens* isolated from stable fly feces attracted stable flies regardless of their sex and reproductive status (Fig. 6), possibly indicating that it also plays a functional role in a foraging context. Genotyping these various *S. marcescens* strains and characterizing their odor profiles may reveal between-strain metabolic differences (Basso et al. 2022) with potential strain-specific odor profiles (Rajendran et al. 2024).

Odor profiles of bacteria may also change in relation to the nutrient source available for metabolism (Basso et al. 2022, Rajendran et al. 2024). *Serratia marcescens* grown on egg-yolk media expressed many enzymes that were not detected when grown on fly egg media or in nutrient broth (Lysyk et al. 2002), probably resulting in different odor profiles. That *S. marcescens* more strongly attracted flies when grown on TSA rather than on BBA (Fig. 5) may be due to different odor profiles *S. marcescens* emitted when metabolizing the nutrients from these two different agar types. Near-identical attractiveness of *S. surfactantfaciens* and *S. marcescens* to stable flies (Fig. 4) is likely due to their near-identical odor profiles (Fig. 7). Whereas some of these odor constituents, such as dimethyl disulfide, dimethyl trisulfide, 2-hexanone, 2-undecanone, and 2-tridecanone, are conserved in *Serratia* congeners, others are species-specific (Weise et al. 2014). As expected, all feces-derived bacteria emitted CO_2_ and NH_3_ gases (Supplementary Table 3). However, CO_2_ serves as an activation cue for stable flies rather than as an attractant (Gatehouse and Lewis 1973, Warnes and Finlayson 1985), and the combined effect of CO_2_ and NH_3_ is not sufficient for explaining the specific attractiveness of *S. marcescens* to gravid females (Hung et al. unpubl. data) or to foraging stable flies. Thus, further investigation is warranted to identify the semiochemicals emitted by *S. marcescens*, but not by other bacteria, that render *S. marcescens* highly attractive to stable flies (Figs 3 and 5) (Hennig et al. 2024, Hung et al. unpubl. data).

In conclusion, we identified seven distinct bacterial species in stable fly feces. Of these, *S. marcescens* and *S. surfactantfaciens* attracted flies but had no synergistic effect, possibly because the odor profile of these two closely related species is very similar. Bacterial attractiveness to foraging flies was dependent upon the bacterial nutrient source, with *S. marcescens* being most attractive to flies when grown on TSA. Further investigation and comparative analyses of *S. marcescens* strains grown on different substrates may aid in the identification of semiochemical attractants. Such attractants could then be used to develop synthetic semiochemical lures to augment the attractiveness of vision-based traps and to enhance their captures of foraging stable flies (Zhu et al. 2016).

## Supplementary Material

tjag084_Supplementary_Data
