# Supplementary material for: Bacteria in stable fly (Diptera: Muscidae) feces inform foraging decisions of conspecific flies
Source: J Med Entomol. 2026 Jun 13;63(3):tjag084. doi: 10.1093/jme/tjag084 (PMC13264395; doi:10.1093/jme/tjag084)
Supplement: tjag084_Supplementary_Data [file tjag084_supplementary_data.pdf]

Supplementary Table 1. Scientific names of the top five sequences in BLAST® producing significant alignments with the 16S-rRNA sequences of each of seven bacteria isolated from stable fly feces.

Isolate	Scientific name	Query cover	E-value	% Identity	Accession #
1	*Serratia surfactantfaciens*	100	0	99.171	NR_169468.1
	Serratia marcescens	100	0	99.102	NR_114043.1
	Serratia marcescens	100	0	99.033	NR_113236.1
	Serratia marcescens	100	0	98.964	NR_041980.1
	Serratia marcescens	100	0	98.964	NR_036886.1
2	*Serratia marcescens*	99	0	98.951	NR_114043.1
	Serratia marcescens	99	0	98.876	NR_113236.1
	Serratia surfactantfaciens	99	0	98.801	NR_169468.1
	Serratia marcescens	99	0	98.801	NR_041980.1
	Serratia marcescens	99	0	98.801	NR_036886.1
3	*Niallia circulans*	100	0	97.475	NR_112632.1
	Niallia circulans	100	0	97.399	NR_104566.1
	Niallia nealsonii	100	0	96.715	NR_044546.1
	Niallia oryzisoli	100	0	96.327	NR_151979.1
	Cytobacillus gottheilii	100	0	96.174	NR_108491.1
4	*Providencia huaxiensis*	100	0	98.815	NR_174258.1
	Providencia rettgeri	100	0	98.724	NR_042413.1
	Providencia vermicola	100	0	98.633	NR_042415.1
	Providencia rettgeri	100	0	98.633	NR_115880.1
	Providencia alcalifaciens	100	0	98.633	NR_115879.1
5	*Myroides odoratus*	100	0	96.447	NR_112976.1
	Myroides odoratus	99	0	95.062	NR_044698.1
	Myroides injenensis	99	0	93.612	NR_134774.1
	Myroides phaeus	100	0	93.406	NR_117475.1
	Myroides pelagicus	100	0	93.256	NR_114110.1
6	*Proteus terrae*	100	0	98.57	NR_149294.1
	Proteus terrae	100	0	98.302	NR_146019.1
	Proteus columbae	100	0	98.213	NR_159332.1
	Proteus alimentorum	100	0	98.034	NR_163665.1
	Proteus mirabilis	100	0	97.945	NR_114419.1
	Providencia rettgeri	100	0	97.639	NR_115880.1

	Providencia rettgeri	100	0	97.639	NR_042413.1
	Providencia vermicola	100	0	97.568	NR_042415.1
	Providencia huaxiensis	100	0	97.425	NR_174258.1
7	*Providencia burhodogranariea*	100	0	97.143	NR_104914.1

Supplementary Table 2. Sources and chemical purity of authentic standards used to compare their mass spectra and retention indices with those of natural headspace volatiles emitted by *Serratia marcescens* and *Serratia surfactantfaciens* grown on trypticase soy agar and nutrient agar, respectively.

**Compounds**	**Source**	**Purity (%)**
isoamyl alcohol	Fisher Chemical[1]	n/a[2]
dimethyl disulfide	Sigma-Aldrich[3]	99
2-hexanone	Sigma-Aldrich[3]	98
2-heptanone	Sigma-Aldrich[3]	98
anisole	Sigma-Aldrich[3]	99
dimethyl trisulfide	Sigma-Aldrich[3]	99
p-methyl anisole	Sigma-Aldrich[3]	99
2-nonanone	Sigma-Aldrich[3]	99
geranylacetone	Sigma-Aldrich[3]	96
2-undecanone	Sigma-Aldrich[3]	98
*Z*6-2-tridecenone	Gries Lab[4]	n/a[2]
2-tridecanone	Sigma-Aldrich[3]	99

[1] Fair Lawn, NJ, USA.

[2] Purity not reported.

[3] St Louis, MO, USA.

[4] *Z*6-2-tridecenone was oxidized from its corresponding alcohol, which was available from a previous project (Gries *et al.*, 2002).

Supplementary Table 3. Measurements (± SEM) of carbon dioxide (CO_2) and ammonia (NH_3) emissions by bacteria isolated from stable fly feces. Three spread plates of each species were incubated 24 h at 30 °C on either trypticase soy agar (TSA) or nutrient agar (NA), before being measured independently (n = 3).

**Species**	**Agar type**	**CO_2 (ppm)**[1]	**NH_3 (ppm)**[2]
Serratia surfactantfaciens	NA	455 ± 7	50 ± 8
Serratia marcescens	TSA	454 ± 4	65 ± 6
Niallia circulans	TSA	452 ± 13	20 ± 2
Providencia huaxiensis	TSA	446 ± 6	90 ± 6
Myroides odoratus	TSA	455 ± 7	81 ± 6
Proteus terrae	NA	445 ± 6	44 ± 2
Providencia rettgeri	NA	442 ± 6	38 ± 2

[1] The ambient CO_2 level was 417 ppm.

[2] The ambient NH_3 level was 0 ppm.
